# A novel methodological approach to understanding the cortical and subcortical effects of aerobic exercise in Parkinson’s disease

**DOI:** 10.3389/fnhum.2025.1657049

**Published:** 2025-10-20

**Authors:** Mandy Miller Koop, Anson B. Rosenfeldt, Visar Berki, Andrew Bazyk, Sara Davidson, Nitesh Singh Malan, Sean Nagel, Benjamin L. Walter, James Y. Liao, Jay L. Alberts

**Affiliations:** ^1^Department of Biomedical Engineering, Research Institute, Cleveland Clinic, Cleveland, OH, United States; ^2^Concussion Center, Neurological Institute, Cleveland Clinic, Cleveland, OH, United States; ^3^Center for Neurological Restoration, Neurological Institute, Cleveland Clinic, Cleveland, OH, United States

**Keywords:** Parkinson’s disease, aerobic exercise, subthalamic nucleus, local field potential, electroencephalogram

## Abstract

**Introduction:**

Aerobic exercise mitigates symptoms of Parkinson’s disease (PD) and may slow disease progression; however, the neural mechanisms underlying these improvements are not well understood. In this study, we discuss the methodology for simultaneously recording local field potentials (LFP) from the subthalamic nucleus (STN), cortical activity using scalp electroencephalography (EEG), and exercise performance metrics during a 40-min aerobic cycling session. Data from a single patient with PD are presented to illustrate the utility, feasibility, and data integrity of the experimental set up.

**Methods:**

The Medtronic Percept™ DBS system was used to record and stream bilateral STN-LFP in the OFF-therapy condition (OFF-DBS and OFF-antiparkinson medications) during a 40-min aerobic exercise session. A 64-channel mobile EEG system recorded cortical data. The neural data streams were synchronized using a TENS device that injected a specified electrical signal into the EEG and LFP recordings. Exercise performance metrics, heart rate, cadence, and power were synchronized with neural data and collected during the exercise session. The study is registered on ClinicalTrials.gov, trial identifying numbers NCT05905302 and NCT05972759.

**Results:**

STN-LFP, EEG, and exercise performance data can be synchronized, recorded for more than 40 min, and analyzed to evaluate how aerobic exercise impacts patterns of cortical and subcortical neural activity.

**Conclusion:**

While exercise positively affects symptoms of PD, the precise effects of exercise on network activity remain unclear. The methods utilized for collecting and analyzing neural (cortical and subcortical) and exercise-related data during a typical bout of aerobic exercise suggest that this approach can be adopted for larger, long-term exercise studies in patients with PD and deep brain stimulation (DBS). The described protocol provides a roadmap for future projects aiming to combine STN-LFP and cortical data to better understand how exercise may alter cortico-basal-ganglia-thalamic dynamics in PD.

## 1 Introduction

Parkinson’s disease (PD) is a heterogeneous, neurodegenerative disorder characterized by four cardinal symptoms: tremor, rigidity, bradykinesia, and postural and gait dysfunction. Excessive beta-band synchrony (13–30 Hz) between the subthalamic nucleus (STN) and the frontal cortex reflects abnormal cortico-basal ganglia-thalamic (CBT) dynamics, which are hypothesized to underlie impaired motor and cognitive functions (Degos et al., 2009; Hammond et al., 2007; Mallet et al., 2008a; Mallet et al., 2008b; Uhlhaas and Singer, 2006). Pathological beta activity, specifically in the STN, can be suppressed with dopamine replacement medication (Tinkhauser et al., 2017) and deep brain stimulation (DBS) (Bronte-Stewart et al., 2009).

In addition to medication and DBS, aerobic exercise is an important component of comprehensive PD management (Osborne et al., 2022). Specifically, cycling has been recognized as a safe and effective intervention to mitigate PD symptoms (Tiihonen et al., 2021), as the ability to cycle often remains preserved even as the disease progresses and gait impairments (e.g., freezing of gait episodes) worsen (Snijders et al., 2012). This preservation may result from distinct motor control dynamics. Strozer et al. reported that gait and cycling involve different oscillatory cortical controls, with cycling being associated with a greater power decrease in the high beta band within the motor and supplemental motor cortices during movement initiation and execution, compared to walking, in healthy controls (Storzer et al., 2016). Cycling, specifically at high cadence, has been shown to mitigate global PD symptomology (Miner et al., 2020; Palmieri et al., 2024; Ridgel et al., 2009; Tiihonen et al., 2021).

Despite the promise of aerobic exercise in mitigating PD symptoms and potentially slowing disease progression (Ridgel et al., 2009; Schenkman et al., 2018; van der Kolk et al., 2019), the neural mechanism(s) underlying these improvements remain poorly understood. Support for exercise-induced changes to the CBT network largely comes from studies evaluating short, discrete movements using small muscle groups with limited degrees of freedom (i.e., finger tapping) (Joundi et al., 2013; Stegemoller et al., 2016) and from studies evaluating very short-duration lower extremity cycling movements (10–45 s) (Bougou et al., 2024; Storzer et al., 2017). Bougou et al. (2024) reported a 15–25% reduction in beta-band activity during 2 s of active and passive cycling. Changes in basal ganglia activity during discrete or short-duration movements provide a rationale to hypothesize that high-intensity aerobic exercise reduces STN hypersynchrony and improves the functionality of the CBT circuit, thereby improving motor function following exercise. However, investigating this hypothesis requires the testing of patterns of cortical and subcortical activity during prolonged bouts of aerobic exercise that meet the current clinical recommendations for PD patients (e.g., ~30 min) (Parkinson's Foundation, 2021). Understanding the underlying neural mechanism(s) of exercise in PD has the potential to refine exercise prescriptions (i.e., frequency, duration, time, and mode) and inform DBS programming.

Streaming and recording cortical and subcortical activity during prolonged aerobic activity present several unique challenges, including the temporal synchronization of STN and cortical recordings, limitations of hardware streaming capabilities, and complex artifact interference. This manuscript details a novel methodology and experimental set up used to simultaneously record STN-LFP, cortical EEG, and exercise performance data during prolonged aerobic exercise in individuals with PD and implanted DBS devices. To the best of our knowledge, this is the first paradigm to continuously record both cortical and subcortical signals during a 40 + min exercise session. To demonstrate the feasibility and data integrity of the experimental setup, data from a single PD patient are presented. The experimental paradigm and associated methods are currently being implemented in a larger clinical trial aimed at systematically investigating the mechanism(s) by which aerobic exercise affects CBT dynamics in individuals with PD.

## 2 Materials and methods

### 2.1 Materials and equipment

**Figure 1:**
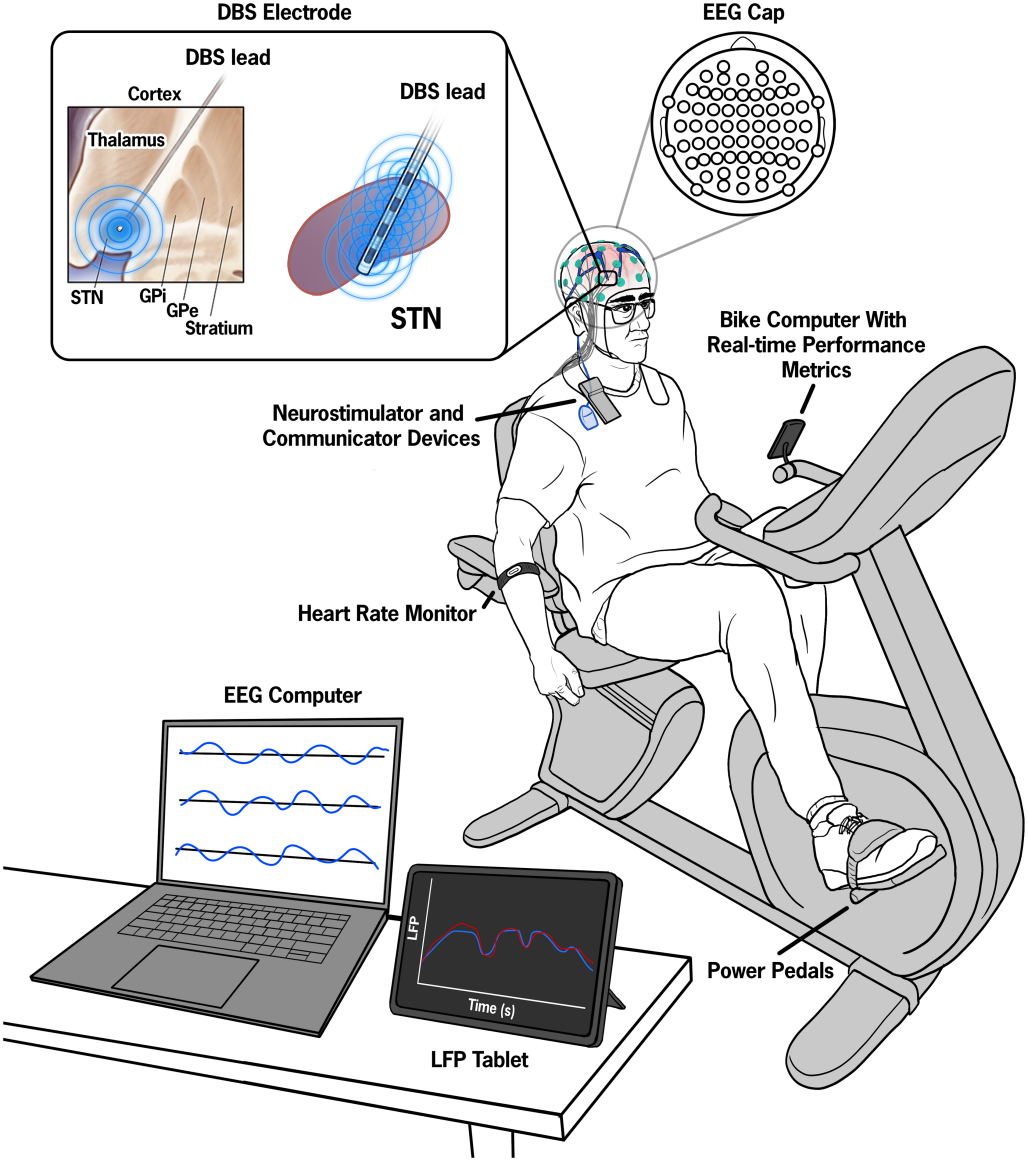
Illustration of the experimental set up. Pre-, during, and post-exercise data from a 64-electrode EEG system were continuously recorded throughout the session and visualized on a control computer. Manual inputs in the control computer denoted key events such as the start and end of the exercise session. The STN-LFP data were streamed to a tablet device in 15-min intervals and synchronized with the EEG data by injecting a known electrical artifact using a superficial TENS unit attached to the wires extending from the IPG and contralateral mastoid.

Three data streams were synchronized, monitored, and collected as part of the experimental paradigm: exercise performance data, STN-LFP data from the STN, and cortical EEG data. The experimental set up is shown in Figure 1.

#### 2.1.1 Cycling equipment

Data from our laboratory suggested that forced exercise (FE), in which cycling cadence is increased by approximately 30% to achieve a rate higher than the participant’s voluntary cadence, may be more beneficial than self-selected cadence cycling in individuals with PD (Miner et al., 2020; Ridgel et al., 2009). In the FE paradigm, a recumbent cycle was equipped with a motor and a custom motor control algorithm designed to mimic a tandem cycle set up, which has been shown to improve motor symptoms in PD. The control algorithm instantaneously responds to the torque exerted on the pedals by the participant, and the motor supplies the minimal torque necessary to maintain the enhanced, constant cadence. Heart rate (Wahoo TICKR FIT armband, Wahoo Fitness, Atlanta, GA, United States), pedaling rate, and power (Garmin Rally XC200 Dual-Sensing Power Meter Pedals, Olathe, Kansas, United States) were transmitted via Bluetooth to a cycling computer (Garmin Edge 1040 Bike Computer, Olathe, Kansas, United States). Heart rate and pedaling metrics were continuously displayed to provide real-time feedback to both the participant and the experimenter.

#### 2.1.2 STN-LFP equipment

Bilateral STN-LFP data were recorded using the Percept DBS system (Medtronic, Minneapolis, Minnesota, United States). The data were collected in 15-min intervals (maximal duration allowed per single-streaming epoch by Medtronic software) using the Indefinite Streaming Mode at a sampling rate of 250 Hz. Signals from six channel pairs, three per hemisphere, were transmitted via Bluetooth to the Medtronic tablet. The adjacent bipolar channels were then reconstructed from the STN-LFP data extracted from the JSON file, resulting in six channels per hemisphere. The extracted channels included STN_0–3_, STN_0–2_, and STN_1–3_, while the reconstructed channels included STN_0–1,_ STN_1–2,_ and STN_2–3_.

#### 2.1.3 Cortical EEG equipment

EEG signals were acquired using the Brain Vision LiveAmp 64 mobile system (Brain Products GmbH, Gilching, Germany). The EEG data were recorded from 64 channels via ActiCap Slim active Ag/AgCl electrodes. The electrodes were embedded in an elastic cap, with electrode positions conforming to the international 10/10 system (Jasper, 1958). Electrode impedances were kept below approximately 20 kΩ. All leads were referenced to the electrode at the FCz position according to the 10/10 international system (Klem et al., 1999). The electrode cables were secured with cable ties near the head to minimize movement during recording. Continuous data acquisition was performed using Brain Products GmbH, Gilching, Germany software at a sampling rate of 500 Hz.

### 2.2 Methods

#### 2.2.1 Participant

The study received approval from the Institutional Review Board of the Cleveland Clinic. Before initiating the study protocol, the participant completed the informed consent process, which included an understanding that the assessment would be conducted in the OFF-medication/OFF-DBS state. The participant was a 71-year-old white man with a 19-year history of PD and a 10-year history of bilateral DBS. He underwent a battery replacement procedure 2 years before enrollment, which upgraded his neurostimulator to facilitate STN-LFP recording. The Percept DBS System was turned OFF 1 h before data collection to ensure an adequate stimulation wash-out period. Clinical ratings during the OFF-medication/OFF-DBS state included an MDS-UPDRS III score of 68 and a Hoehn and Yahr stage of IV, indicating significant disability. The MDS-UPDRS III postural instability and gait dysfunction (PIGD) score was 18 (sum of items 3.9–3.13; range 0–20; higher score indicates worse impairment) (Makkos et al., 2018), indicating significant PIGD symptoms in the OFF-medication/OFF-DBS state (Goetz et al., 2008).

#### 2.2.2 Data synchronization and alignment

The EEG computer served as the central synchronization source. Data synchronization was achieved through (1) electrical noise injected into both the EEG and DBS data streams and (2) manual inputs denoting the start, warm-up, cool-down, and cessation of the exercise session. Synchronization between the STN-LFP and EEG data streams was achieved by injecting a known electrical artifact into both streams (Thenaisie et al., 2021) using a commercially available TENS device (Electrostimulator Device, BioMedical Life Systems, Inc., Vista, California, United States). Two adhesive disk surface electrodes (Natus Inc., Middleton, Wisconsin, United States) were placed—one at the extension cable insertion point on the implantable pulse generator (IPG) and the other on the contralateral mastoid process. Each time the STN-LFP data stream was initiated, multiple 0.5 s TENS bursts (250 μs, 80 Hz, 1.5 mA) were injected into the neural data streams to provide a common alignment signal.

Alignment of the STN-LFP and EEG data was performed in MATLAB (The MathWorks Inc., Natick, MA, United States). The EEG signal was first downsampled to match the STN-LFP sampling frequency of 250 Hz. Both EEG and STN-LFP data were normalized to their respective maximum values and then filtered using a fourth-order Butterworth bandpass filter between 60 and 100 Hz to isolate the TENS artifact. Cross-correlation analysis (using MATLAB’s xcorr function) was performed to determine the offset and align the data streams by minimizing the error between the TENS artifact signals recorded in both EEG and STN-LFP data streams.

#### 2.2.3 Experimental procedure

Following the preparation of the EEG cap, the participant mounted the recumbent FE cycle. A 1-min baseline recording of EEG and STN-LFP data was captured during quiet sitting, with eyes closed on the stationary cycle. Consistent with current PD aerobic exercise recommendations (Parkinson's Foundation, 2021), the participant engaged in a 40-min exercise session, which consisted of a 5-min warm-up, a 30-min main exercise set, and a 5-min cool-down on the FE cycle. The warm-up and cool-down cadence was set to 50 RPM, while the main exercise set was performed at an FE cadence of 60 RPM (approximately a 30% increase over the participant’s voluntary cadence of 35–40 RPM). After the exercise session concluded, a 5-min post-exercise rest period was recorded with eyes closed.

#### 2.2.4 General data processing methods

For both STN-LFP and EEG data, average power spectral densities (PSDs) were calculated on non-overlapping 1-min blocks for each channel using Welch’s method with a 1-s Hanning window and 50% overlap. The mean power for specific frequency bands was calculated as the area under the curve (AUC) of the linear power spectrum. The frequency bands were defined as follows: delta (1–4 Hz), theta (4–8 Hz), alpha (8–12 Hz), and beta (13–35 Hz). The primary endpoint for this feasibility study was a change in beta-band power (13–35 Hz), presented as means (±standard deviation). Time-frequency spectrograms were computed for frequencies from 1 to 40 Hz using a multitaper convolution method with a 4-s Hanning taper window in the FieldTrip toolbox (Oostenveld et al., 2011). For visualization, spectrogram power was converted to decibels (dB) and normalized by subtracting the mean power of the pre-exercise rest segment. To visualize changes in beta power over time, minute-by-minute beta power (AUC) was calculated and normalized to the mean beta power from the pre-exercise rest segment.

#### 2.2.5 STN-LFP preprocessing and channel selection

The STN-LFP time-domain data, with magnitudes measured in microvolts peak (uVp), were downloaded as a JSON file for offline analysis in MATLAB. For each hemisphere, the LFPs were screened for ECG artifact using a validated QRS template subtraction algorithm, as ECG contamination is a well-known artifact resulting from the location of the implantable pulse generator (IPG) (Neumann et al., 2021; Sanger et al., 2025). To prevent the removal of genuine neural signals due to low-level ECG contamination, ECG artifacts were subtracted from the LFP signal if the power at 8 Hz decreased by more than 2 dB/Hz. This threshold was informed by Medtronic’s adaptive DBS (aDBS) paradigm, where 8 Hz is the lowest frequency of interest used and is most impacted by ECG artifact (Sanger et al., 2025). In this dataset, none of the LFP signals met this criterion; thus, the raw LFP data were utilized for subsequent analysis. Specifically, the channel with the highest mean beta power during the pre-exercise resting state was selected for all subsequent analysis and is referred to as right or left STN.

#### 2.2.6 EEG preprocessing

All EEG preprocessing was conducted using custom MATLAB scripts leveraging the EEGLAB toolbox (Delorme and Makeig, 2004). First, 60 Hz line noise was removed using the CleanLine algorithm. The data were then band-pass filtered between 1 and 41 Hz and resampled to 250 Hz. Artifact subspace reconstruction (ASR) was applied to the EEG channels to correct for non-stationary noise, using a BurstCriterion of 4 standard deviations, a FlatlineCriterion of 4 s, and a ChannelCriterion of 0.85 relative to the neighboring electrodes (Chang et al., 2020; Delorme and Makeig, 2004). Any channels removed by ASR were subsequently interpolated using a spherical spline method (Delorme and Makeig, 2004). Following ASR, independent component analysis (ICA) was performed using the runICA algorithm (Bell and Sejnowski, 1995) with 40 principal components. Similar to other studies, components identified by ICLabel (Pion-Tonachini et al., 2019) as muscle, eye, or other artifacts with a probability greater than 70% were removed (Chen et al., 2024; Delorme, 2023; Pion-Tonachini et al., 2019). Finally, a surface Laplacian filter was applied to enhance spatial resolution (Cohen, 2014; Perrin et al., 1989). The cleaned data blocks were then utilized for the final analysis.

## 3 Results

### 3.1 Local field potential data

**Figure 2:**
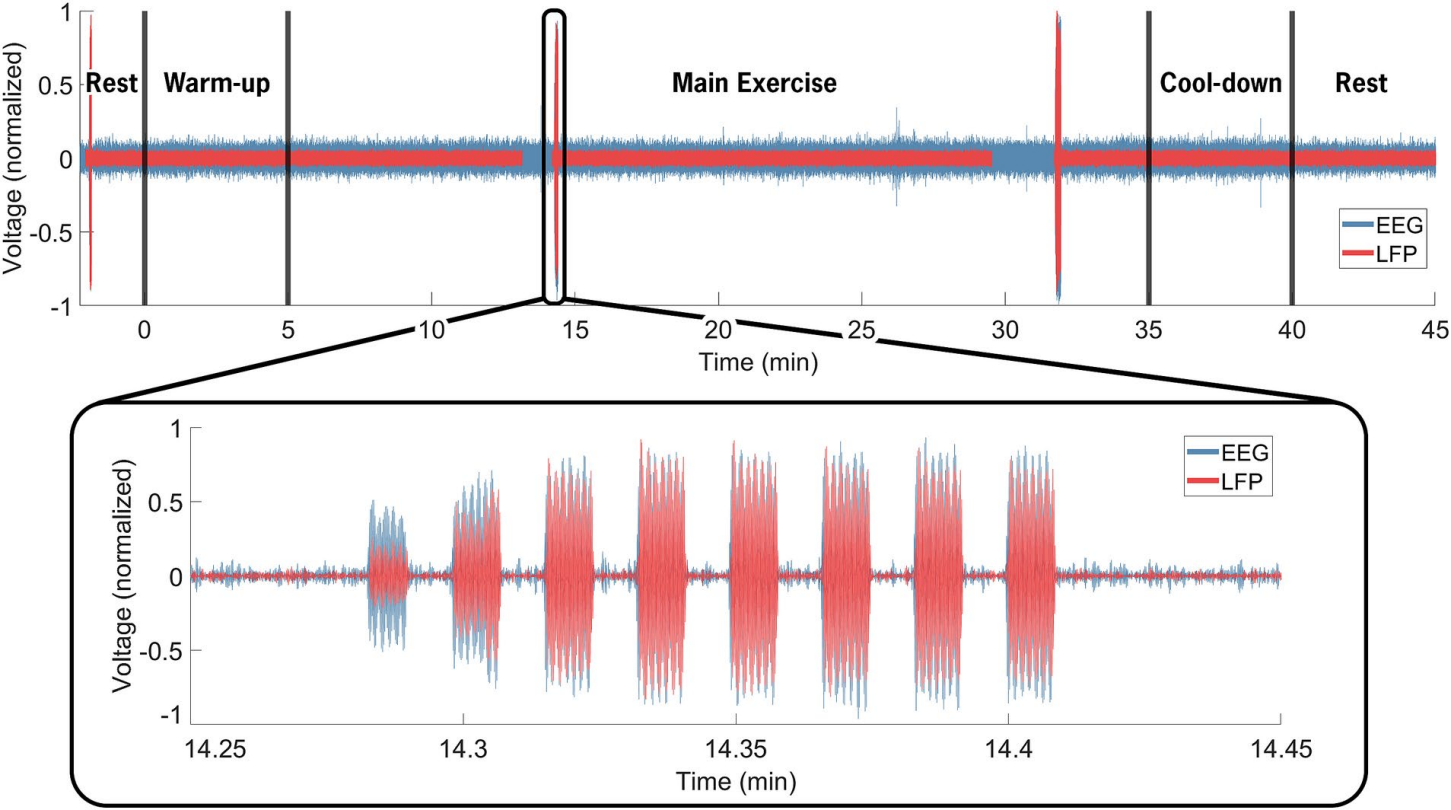
Neural data synchronization process. The EEG computer served as the control computer. Manual inputs denoted the cycling start and stop times (black vertical line at 0 and 40 min). The insert highlights the electrical artifact injected via the TENS device, delivered through electrodes placed near the IPG on the extension cable and on the contralateral mastoid. The artifact was visible in both the EEG (blue) and STN-LFP (red) data streams and facilitated precise temporal alignment between the data streams, resulting in an average 12.3 ms of error across all trials. Over the entire 40-min exercise and data recording session, bilateral STN-LFP data were recorded for 90% (36 min, 7 s/40 min) of the total time, demonstrating the feasibility of prolonged STN recording during exercise.

**Figure 3:**
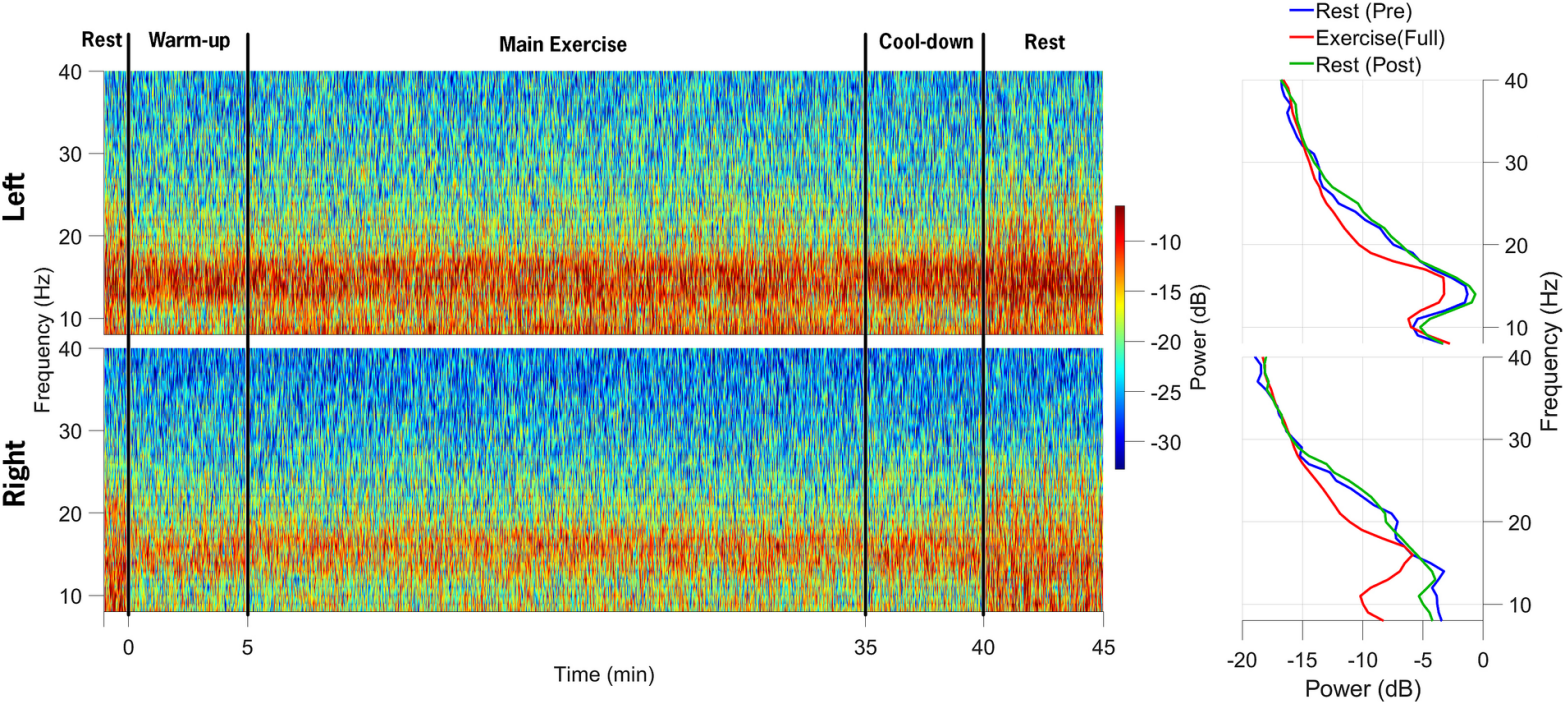
Spectrograms of local field potential (STN-LFP) data from the left STN and right STN electrodes are shown in the top and bottom rows, respectively. The data collection session was divided into five distinct phases: 1-min rest before exercise initiation, 5-min warm-up, 30-min main exercise period, 5-min cool-down, and 5-min post-exercise rest. The power spectral density plots on the right indicate a decrease in alpha and beta-band power during the main exercise period (red) compared to the pre-exercise (blue) and post-exercise (green) periods.

Data synchronization across the STN-LFP and EEG data streams was successfully achieved (Figure 2). Figure 3 illustrates spectrogram and PSD data from the left and right STN pre-, during-, and post-exercise. Bilateral STN-LFP data were recorded for 90% (36 min, 7 s/40 min) of the total exercise duration, demonstrating the feasibility of prolonged STN recording during realistic exercise sessions.

**Figure 4:**
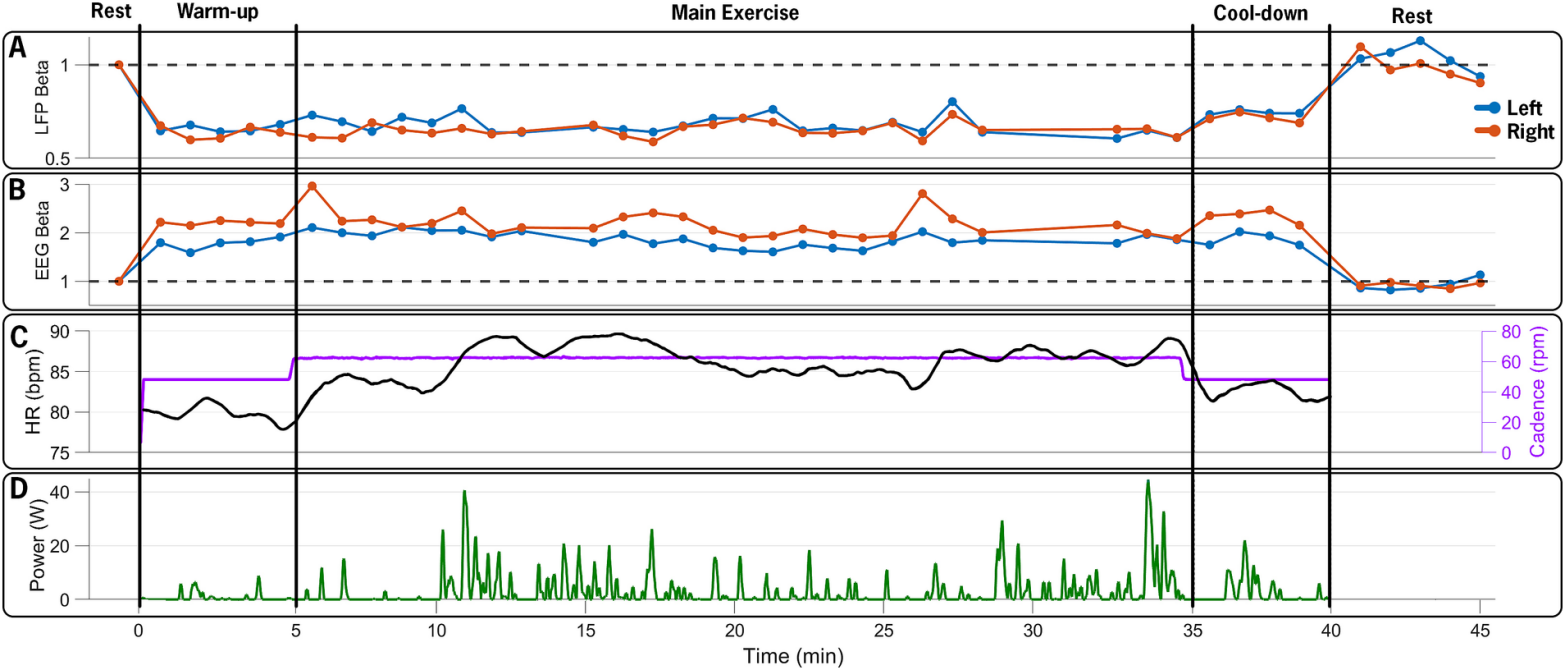
Time series of neural and exercise data over the 45-min data collection session. **(A)** Normalized beta-band power in 60-s epochs from left (blue) and right (red) STN contacts, **(B)** normalized 60-s beta-band power epochs from EEG electrodes over the left (C3, blue) and right (C4, red) M1, **(C)** heart rate (black) and pedaling cadence (purple), and **(D)** pedaling power output during the 40-min exercise period. The FE bike maintained a steady cadence of 60 rpms, which was approximately 30% greater than the participant’s self-selected cadence. The participant’s heart rate and power increased, reflecting the increased effort during the main exercise set. The figure illustrates the capability of the design paradigm to capture and synchronize multiple distinct data streams (STN-LFP, EEG, and exercise performance) over a prolonged bout of aerobic exercise.

Beta-band power from the channel with the highest resting-state beta power was normalized to the pre-exercise resting-state beta-band power in 60-s epochs. Compared to the resting-state baseline, the average beta-band power in the LFP channels decreased bilaterally, from 3.84 (0.38) μV^2^/Hz at baseline (rest-pre) to 2.74 (0.24) μV^2^/Hz (71% reduction) for the left STN, and from 2.47 (0.2) μV^2^/Hz to 1.68 (0.12) μV^2^/Hz (68% reduction) for the right STN at the mid-point (~20-min point) of the FE session (Figure 4). One minute post-exercise, the average beta-band power initially rebounded above baseline values bilaterally to 3.96 (0.35) μV^2^/Hz (103% increase) and 2.71 (0.2) μV^2^/Hz (110% increase) for the left and right STN, respectively, before declining back to approximately baseline values.

### 3.2 Cortical EEG data

**Figure 5:**
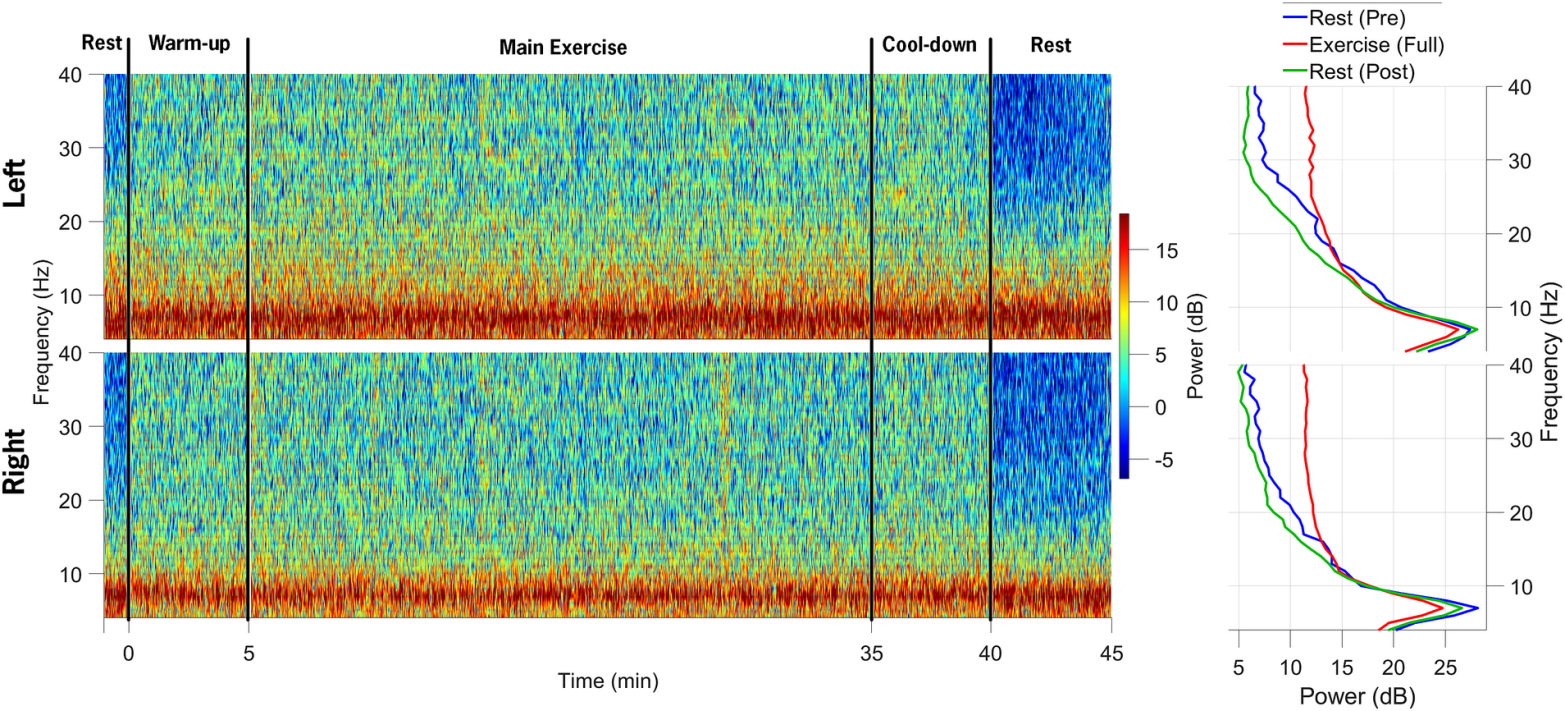
Spectrograms of EEG data from the left and right M1 electrodes are shown in the top and bottom rows, respectively, across the following segments of the data collection session: (1) 1-min rest before exercise, (2) 5-min warm-up, (3) 30-min main exercise period, (4) 5-min cool-down, and (5) 5-min post-exercise rest. The power spectral density plots on the right demonstrate an increase in beta-band power during the main exercise (red) compared to both pre-exercise (blue) and post-exercise (green) periods.

Figure 5 shows a spectrogram of the EEG data from the left and right primary motor cortex (M1; channels C3 and C4). Across all trials, 94% of the EEG data were retained after post-processing and included in the analysis. Beta-band power from the left and right M1 channels (C3 and C4) was normalized to the pre-exercise resting-state beta-band power in 60-s epochs. Compared to the baseline (rest-pre), the average beta-band power increased from 218.24 (15.19) μV^2^/Hz to 368.23 (20.68) μV^2^/Hz (169% increase) for the left M1 and from 148.77 (10.99) μV^2^/Hz to 305.17 (15.68) μV^2^/Hz (205% increase) for the right M1 at the mid-point of the FE session (~20-min mark). One minute after exercising, the average beta-band power (Figure 4) decreased relative to the baseline, falling to 3.96 (0.35) μV^2^/Hz (86% of the baseline) and 2.71 (0.2) μV^2^/Hz (91% of the baseline) for the left and right M1, respectively.

## 4 Discussion

While exercise has been shown to positively impact symptoms of PD, the underlying mechanisms contributing to these improvements remain poorly understood, in part, due to the difficulty of collecting and characterizing neural data in humans during physical activity. The protocol described in this manuscript, to our knowledge, is the first to capture concurrent STN-LFP and cortical EEG (64 channels) during a prolonged bout of aerobic exercise. The methodology is replicable and adaptable for systematically evaluating CBT network activity in individuals with PD and implanted DBS systems, leveraging novel capabilities of the Percept DBS device. Key challenges included synchronizing STN-LFP and EEG data streams, streaming duration limitations of the Percept DBS device, and artifact interference during the prolonged exercise task. The initial results indicate that neural and exercise performance data can be successfully synchronized, collected, and analyzed, providing proof-of-concept that aerobic exercise may be capable of altering CBT network activity. The success of the experimental and data collection protocol offers a viable roadmap for future exercise projects and studies aiming to understand the potential neural mechanisms underlying specific motor, cognitive, and cognitive-motor symptoms and dysfunction in individuals with PD.

Data from healthy adults indicate that exercise alters cortical activity; specifically, 4 weeks of cycling reduced cortical alpha and beta power compared to a no-exercise control group (Ludyga et al., 2016). Consistent with the neural efficiency hypothesis (Neubauer and Fink, 2009), the cortical changes were most pronounced at 120 RPM in individuals who trained at higher cadences (Ludyga et al., 2016). In PD, accumulating evidence suggests that high-intensity exercise is more effective than lower-intensity exercise for symptom mitigation and potential disease modification (Ahlskog, 2011; Schenkman et al., 2018). Specifically in the context of cycling, our previous research indicated that cycling at a higher cadence resulted in improved motor outcomes (Ridgel et al., 2009) and improved thalamo-cortical connectivity, as measured by functional magnetic resonance imaging (fMRI) (Shah et al., 2016), compared to cycling at lower cadences in individuals with PD, suggesting that higher-intensity exercise may elicit a more robust neurophysiological response in individuals with PD. Recent metabolic connectivity data obtained from a mouse model showed that aerobic exercise enhanced CBT network activation and strengthened connectivity between the basal ganglia and cortical regions over a 6-week period (Wang et al., 2023). While basal ganglia dysfunction in PD disrupts both subcortical and cortical circuitry (Wichmann and DeLong, 1996), it is plausible that the neural efficiency hypothesis still applies, albeit in a blunted form. Supporting this, preliminary data from nine PD patients with the Percept DBS showed an increase in power in the aperiodic metrics of the dorsal STN following a 4-week exercise intervention, despite no significant changes after acute cycling (Joshi et al., 2025). Together, these findings suggest that exercise can induce changes in the CBT network, although the extent and duration of these changes are not known. Thus, it is reasonable to pursue a systematic investigation of the neural mechanism(s) underlying exercise in PD to further understand the impact of exercise on brain function and optimize exercise prescription for PD patients.

The primary purpose of this manuscript was to describe the methodology associated with collecting cortical and subcortical data during a realistic bout of aerobic exercise. Although successful, some limitations exist. This report presented data from a single participant as a proof of concept; thus, the neural findings are preliminary. Several approaches were utilized to minimize the noise and artifacts in the LFP and EEG signals; however, residual noise may still have influenced the results. Although not observed in this dataset, prolonged LFP recordings using the Percept DBS system can result in signal dropout. To avoid this issue, the LFP recordings were limited to ~15-min durations, and the Medtronic communicator was hardwired to the tablet (Medtronic, 2024). The termination and initiation of trials throughout the 40 + min session resulted in approximately 10% data loss in the LFP recordings compared to the EEG, but it also prevented large blocks of data loss. Finally, it remains unclear how acute, transient neural and symptomatic changes from a single exercise session translate to sustained improvements with chronic exercise. To address this limitation, a larger clinical trial is underway, which aims to evaluate the impact of aerobic exercise on a larger cohort (*N* = 25) of individuals with PD. The study includes an FE arm and a voluntary rate arm, which may provide insights into the impact of intensity and cadence on periodic and aperiodic measures (Zhang et al., 2021) of the CBT network. The methodology offers a framework for investigating other potential factors affecting exercise outcomes, such as the impact of medication. Extending post-exercise recording durations may provide insights into the long-term impact of aerobic exercise.

The methodology described in this report demonstrates that systematic investigation of exercise effects on the CBT network is feasible. Medtronic’s “sensing” DBS devices, such as the Percept, provide a unique opportunity to monitor basal ganglia function (Farokhniaee et al., 2024; Quinn et al., 2015; Syrkin-Nikolau et al., 2017), which was previously accessible only through externalized leads immediately following DBS surgery. The initial data confirm the feasibility of collecting continuous neural and performance data from an individual with advanced PD in the OFF-medication/OFF-DBS state. This data collection and processing approach is being utilized in an ongoing project to determine the impact of an acute bout of aerobic exercise on CBT function. A standardized approach to the data collection and analysis of data gathered using the Medtronic Percept platform will ultimately facilitate consistency, accuracy and reliability of recordings. The technology and methods presented in this report pave the way for using STN-LFP and EEG recordings while PD patients engage in exercise or activities that elicit or trigger motor symptoms (e.g., dual-tasking, freezing of gait).

## Data Availability

The raw data supporting the conclusions of this article will be made available by the authors, without undue reservation.
